# Driving clinical excellence through a management driven framework for integrating nuclear medicine into hospital ecosystems

**DOI:** 10.3389/fnume.2026.1837305

**Published:** 2026-06-15

**Authors:** Chuangyan Zhai

**Affiliations:** 1 Center for Orthopedic Surgery, The Third Affiliated Hospital of Southern Medical University, Guangzhou, China; 2 Shanghai Key Laboratory of Molecular Imaging, Shanghai University of Medicine and Health Sciences, Shanghai, China

**Keywords:** hospital administration, interdisciplinary collaboration, nuclear medicine, total performance index (TPI), value-based health care

## Abstract

**Purpose:**

Nuclear medicine (NM) often functions as an isolated diagnostic island within hospitals, resulting in the underutilization of high-value molecular imaging resources despite their significant clinical potential. This paper proposes a conceptual management-driven framework, termed the Management-Driven Integration Loop, to break down these silos and integrate NM into the broader hospital ecosystem to potentially enhance clinical decision-making and operational efficiency.

**Methods:**

A comprehensive integration framework was developed based on four strategic pillars: strategic resource allocation, process re-engineering, performance leverage, and brand building. The framework introduces specific administrative interventions, including the establishment of satellite workstations to improve clinical proximity, the creation of a Clinical-NM Liaison role to bridge interdisciplinary gaps, and the implementation of a multidimensional Total Performance Index (TPI) to align incentives.

**Framework components:**

Integration is envisioned through protocol-based clinical pathways embedded in electronic health records and structured Multidisciplinary Team (MDT) collaboration, ensuring NM expertise may inform treatment planning. The framework incorporates a continuous feedback loop to track clinical impact, including diagnostic upstaging and the avoidance of unnecessary procedures. By proposing a shift from volume-based metrics to the TPI, administrators can weight qualitative clinical contributions and interdisciplinary collaboration alongside traditional procedural throughput.

**Conclusions:**

Transforming nuclear medicine into a central strategic asset requires deliberate administrative intervention. While implementation depends on overcoming institutional, technical, and regulatory barriers, this management-driven integration loop aims to bridge the gap between technological potential and clinical impact, improve patient outcomes through precision diagnostics, and strengthen institutional positioning as centers of excellence.

## 1 Introduction

Molecular imaging technologies, including PET/CT and SPECT/CT, have transformed modern medicine by integrating anatomical and functional information to provide more precise, patient-specific insights into disease processes (1). The emergence of PET/MR and the rapid development of theranostics have further expanded the clinical scope of the field (2, 3). These advancements have the potential to be essential across multiple specialties, particularly in oncology, cardiology, and neurology, where molecular imaging can enhance diagnostic accuracy, disease characterization, and treatment planning (4). Yet in many hospitals, nuclear medicine suffers from a persistent problem: despite significant investment in high-value molecular imaging resources, which often represents the largest capital expenditure in an institution, its clinical potential may remain underutilized (5). Nuclear medicine frequently functions as a “diagnostic island,” isolated physically, operationally, and intellectually (6). Imaging findings are often treated as supplementary data rather than central drivers of treatment planning (7). As a result, opportunities to guide personalized therapy, avoid unnecessary procedures, and optimize resource use could be overlooked.

This isolation is rarely a reflection of the skills or dedication of nuclear medicine specialists; instead, it reflects structural and administrative challenges. Departments often operate in functional silos, which reinforces the physical isolation of the “diagnostic island”. Interdepartmental communication is often inconsistent, and incentives frequently fail to reward collaborative clinical impact (8). Since relying on informal collaboration alone is insufficient, administrators play a crucial role in addressing these systemic barriers (1). Proactive governance, implemented through clear policies, workflow integration, and performance accountability, is proposed as a necessary step to transform nuclear medicine from a peripheral service into a central clinical and strategic asset.

Drawing a parallel to pathology, which has long advocated for its role as the definitive diagnostic foundation of medicine, we argue that nuclear medicine must similarly be recognized as a “navigational hub” for precision medicine at the molecular level. To address these systemic barriers and bridge the gap between technological potential and institutional practice, this paper serves as both a conceptual commentary and an administrative blueprint.

**Figure 1:**
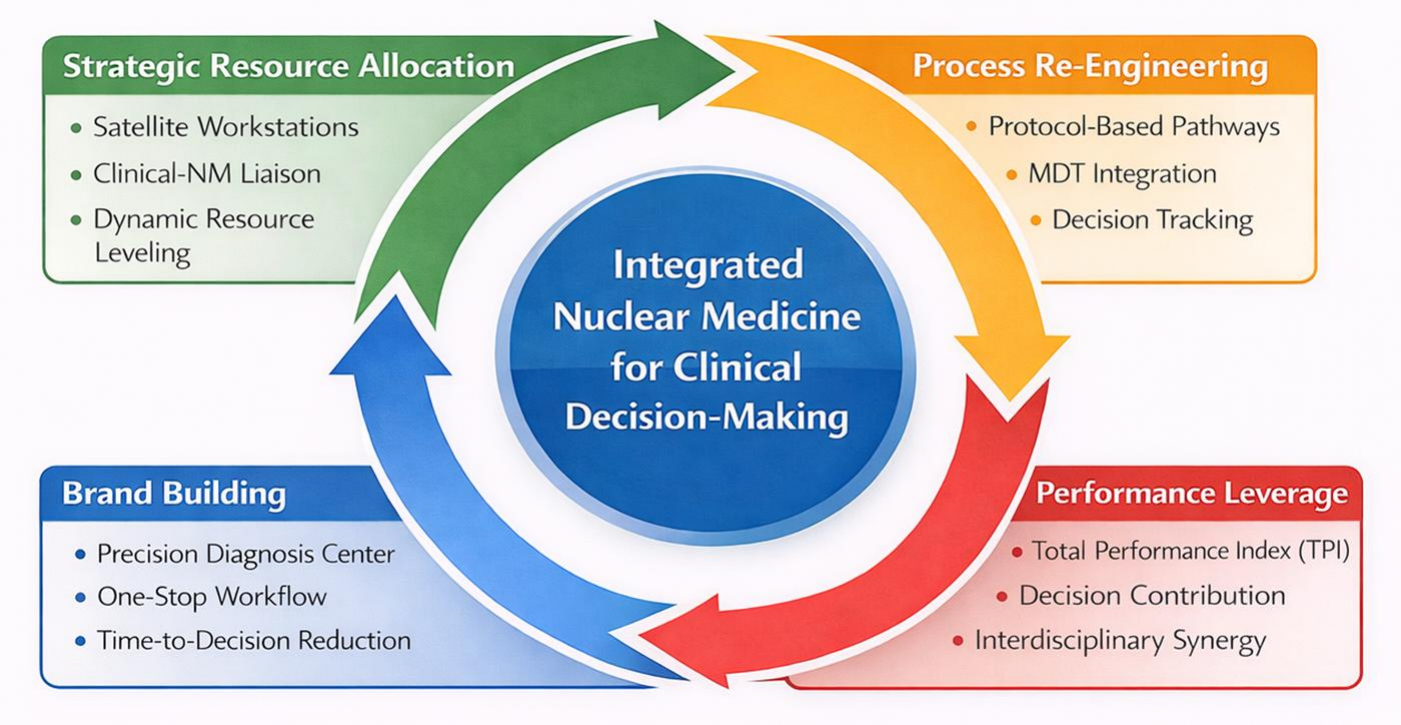
The management-driven integration loop for nuclear medicine.

Accordingly, the proposed framework is centered on four pillars, forming a structured Management-Driven Integration Loop (Figure 1): strategic resource allocation aims to ensure that equipment and staff are positioned to maximize clinical value; process re-engineering suggests establishing protocol-based clinical pathways and structured multidisciplinary collaboration; performance leverage explores aligning incentives using the TPI; and brand building seeks to elevate nuclear medicine from a supportive service to a center of excellence. Collectively, these components are designed to continuously reinforce each other to maximize the long-term Return on Mission (ROM), ultimately unlocking the potential of molecular imaging to inform critical decisions and drive institutional excellence.

## 2 Strategic resource allocation: constructing the infrastructure

### 2.1 Physical integration: moving beyond isolation with satellite workstations

Nuclear medicine departments are often physically marginalized due to strict radiation shielding requirements, frequently placed in basements or remote areas (9). This spatial isolation can exacerbate the “diagnostic island” problem, as it creates both physical and psychological barriers that may limit real-time consultation with clinicians (10).

To address this structural isolation and improve clinical proximity, larger tertiary hospitals could consider establishing satellite workstations within core clinical hubs, such as oncology wards, cardiology centers, and neurology units. These nodes are envisioned to function as interdisciplinary spaces where specialists participate in clinical rounds. While administrators might provide office space to support these “embedded” specialists, the feasibility of this model may depend on the institution's scale and available staffing levels. By moving toward an “imaging-to-bedside” model, hospitals aim to reduce consultation delays and ensure molecular insights inform treatment at an earlier stage. This approach is expected to strengthen interdisciplinary collaboration, promote a culture of proactive diagnostics, and maximize the value of high-capital equipment.

### 2.2 Specialized personnel: the clinical-nuclear medicine (NM) liaison

Physical proximity alone may not be sufficient. Hospitals should consider the need for specialized personnel to bridge interdisciplinary gaps. The Clinical-NM Liaison is a proposed formal role designed for this purpose. Unlike Nuclear Medicine Physicians who primarily focus on volume-based reporting, the Clinical-NM Liaison is intended to serve as the central clinical coordinator, a role analogous to specialized coordinators in oncology multidisciplinary teams (MDTs), ensuring that molecular data is synthesized with genomic and pathological findings to provide a cohesive “biological map” for the treating physician (11).

To be effective, the liaison would proactively review complex cases and suggest adjustments to scanning parameters, such as tracer selection, imaging timing, or delayed scans, to ensure that results are directly actionable for surgeons or oncologists. While this role requires administrative empowerment to coordinate interdepartmental conferences, candidates could be drawn from senior physicians undergoing targeted cross-training in clinical pathways, supported by dedicated time allocations and direct HIS/PACS workflow integration to mitigate burnout. Its implementation may be scaled according to the hospital's workforce capacity, potentially starting as a shared responsibility in smaller institutions. By institutionalizing this role, nuclear medicine has the potential to evolve from a passive diagnostic service into a proactive clinical consultancy, ensuring high-value imaging is fully understood, communicated, and applied in patient care.

### 2.3 Technological support: internet of things (IoT)-driven platforms and dynamic resource leveling

Maximizing the utilization of high-cost equipment could benefit from administrative oversight combined with technological support. Cloud-based Internet of Things (IoT) monitoring platforms can provide real-time visibility into scanner usage, patient flow, and appointment backlogs across multiple hospital sites. Administrators could leverage this data to implement dynamic resource leveling.

Unlike chronological booking, this proposed system prioritizes cases based on clinical value. For example, when a primary cancer center's scanner is overbooked, routine follow-up scans could be redirected to underutilized satellite scanners, while urgent, high-complexity cases are scheduled on the most advanced, high-resolution equipment. This approach is designed to improve patient access, reduces waiting times, and prevents equipment idling. Administratively, it aims to ensure that the hospital's most critical clinical needs are matched with the appropriate resources, potentially providing a measurable operational return on investment (ROI) and ensuring that expensive imaging assets deliver maximum clinical and institutional impact.

## 3 Process re-engineering: institutionalizing interdisciplinary collaboration

### 3.1 Protocol-based clinical pathways

To overcome the limitations of ad-hoc referral model, it is suggested that administrators explore the development of protocol-based clinical pathways to ensure diagnostic consistency (12). These pathways could identify key clinical inflection points, such as initial staging for high-risk prostate cancer or therapy response assessment in lymphoma, where PET/CT evaluations including PSMA or FDG scans are mandated by hospital-wide Standard Operating Procedures (SOPs).

By proposing the inclusion of these pathways in the electronic health record (EHR) system, molecular imaging could become a structured rather than optional part of the diagnostic journey. While such integration aims to standardize care, it should be designed to maintain clinical flexibility, allowing physicians to adapt these pathways based on individual patient needs.

### 3.2 Structured multidisciplinary team (MDT) integration

Traditional MDTs often suffer from imaging fragmentation, where nuclear medicine results are treated as isolated data points with limited influence on treatment planning. To address this, this framework proposes a structured integration model where nuclear medicine specialists play a more active role in diagnostic discussions. Instead of merely reporting findings, nuclear medicine physicians are encouraged to provide integrative insights that synthesize imaging with therapeutic possibilities. By formalizing the inclusion of molecular perspectives in final treatment recommendations, hospitals aim to transition nuclear medicine from a supportive service to an active participant in precision medicine.

### 3.3 MDT feedback and decision-tracking loop

Even well-structured MDTs can fail without accountability. To address this, administrators could consider establishing an MDT feedback and decision-tracking loop, integrated into the Hospital Information System (HIS), to monitor performance across multiple strategic dimensions. This loop systematically captures measurable outcomes to assess clinical, operational, and economic impacts. The potential focus of this mechanism is to explore the quantification of clinical impact by tracking the adoption rate of nuclear medicine-informed recommendations, particularly in cases where molecular imaging may lead to significant diagnostic upstaging, downstaging, or a fundamental shift in the treatment plan (13). Beyond clinical outcomes, this conceptual feedback system could monitor key operational efficiency metrics, such as the Average Length of Stay (ALOS). Theoretically, by providing earlier and more definitive staging, nuclear medicine has the potential to accelerate the transition from initial clinical suspicion to targeted therapy, thereby potentially reducing redundant hospital days and optimizing bed turnover.

Furthermore, this loop aims to translate diagnostic precision into measurable economic value by monitoring the total cost of care. By identifying occult metastases or accurately predicting non-response to expensive pharmacological interventions, specialists could enable the clinical team to avoid unnecessary invasive procedures and ineffective treatments. Crucially, the resulting data on clinical ROI and equipment utilization could be fed back into the Strategic Resource Allocation phase (Section 2). This strategy for “back-feeding” is designed to allow administrators to justify the dynamic redeployment of satellite workstations or liaison personnel to the most high-impact clinical areas, completing the Management-Driven Integration Loop. By bridging this information gap, administrators could ensure that diagnostic precision not only informs individual patient care but also supports long-term institutional efficiency and resource optimization.

An illustrative example of this framework can be considered in the management of high-risk prostate cancer. Under the proposed integration model, eligible patients could be identified through protocol-based pathways embedded within the EHR, followed by coordinated PSMA PET/CT evaluation before MDT discussion. Through the involvement of the Clinical-NM Liaison, who assists in integrating imaging insights with clinical staging, and structured MDT integration, molecular imaging findings may support earlier treatment planning, reduce unnecessary procedures, and shorten the Time-to-Decision (TTD). By avoiding futile interventions, this adaptive process could help translate operational efficiency into an enhanced ROM, and the resulting clinical value could subsequently be logged within the HIS to update the nuclear medicine physician's Decision Contribution (D) variable within the TPI framework, thereby closing the management loop. Although this example is illustrative rather than derived from prospective pilot data, it offers a practical context for understanding how administrative coordination, specialized personnel, and molecular imaging could function synergistically within the proposed Management-Driven Integration Loop.

## 4 Performance leverage: aligning incentives with clinical value

### 4.1 From volume to value: the multidimensional evaluation model

The traditional “fee-for-service” or volume-based compensation model can be a significant deterrent to interdisciplinary collaboration, as time spent in multidisciplinary team (MDT) meetings often yields no direct financial return for the nuclear medicine department. To address this structural misalignment, hospital administrators could consider implementing a conceptual TPI framework to redefine the value contribution of nuclear medicine physicians. Unlike conventional pathways centered on high-volume clinical departments, this framework leverages administrative governance to establish molecular imaging as an active informational hub. Accordingly, the index could be mathematically formulated as follows:

$$TPI\;=\;w_{1}Q+w_{2}D+w_{3}S$$

In this model, Q represents traditional quantitative output, such as scan counts or Relative Value Units (RVUs). D represents Decision Contribution, which aims to capture validated impacts on clinical staging or treatment plan changes. S represents Synergy and Research, such as joint multidisciplinary publications or clinical grants.

The core of administrative power lies in the strategic adjustment of the weighting coefficients (w). During the initial phase of integration, administrators could strategically elevate the weights of w_2_ and w_3_. This is intended to suggest a clear institutional signal: the hospital values active clinical consultation and collaborative innovation as much as, if not more than, technical imaging volume. This shift encourages physicians to move away from “report mills” and toward becoming high-value clinical consultants. Notably, these weights should be adjusted dynamically based on the specific strategic phase and resources of the institution. For example, an initial allocation of 0.4 for w_1_ (Quantity), 0.4 for w_2_ (Decision), and 0.2 for w_3_ (Synergy) could potentially facilitate a transition from volume-centered to value-centered governance.

### 4.2 Quantitative decision weighting in practice

To ensure that the D (Decision Contribution) variable remains as objective as possible, this framework proposes integrating it with the MDT feedback-tracking system established in Section 3. For instance, performance points could be awarded when a nuclear medicine physician's interpretation leads to the avoidance of an unnecessary invasive procedure or the identification of occult metastases that alter the surgical approach. To further operationalize this, the D variable can be categorized into measurable tiers, such as management changes (e.g., palliative to curative intent), diagnostic refinements, and procedure avoidances. To safeguard the integrity of this metric, such contributions should be documented within the HIS or verified by an interdisciplinary audit. This administrative “carrot” is intended to encourage physicians to invest time in pre-meeting preparations and deep clinical engagement, effectively transforming the MDT from a burdensome task into a high-value professional activity. To support operational consistency and validation before active implementation, baseline simulations could be conducted using retrospective data to calibrate the weighting coefficients (w_1_, w_2_, w_3_). Furthermore, a joint interdisciplinary audit committee could be established to periodically review the logged decisions, helping to standardize the evaluation process and mitigate subjective scoring bias.

### 4.3 Joint research funding: breaking budget silo

Beyond individual performance, the administration should consider establishing interdisciplinary strategic seed grants. These funds would be administratively protected and can only be accessed through joint applications from nuclear medicine and at least one other clinical department, such as a partnership between nuclear medicine and urology. By providing matching funds and dedicated research coordinators for these projects, the administration aims to lower the collaboration tax often felt by busy clinicians. This investment not only seeks to drive high-impact translational research but also fosters a culture of mutual dependency. As these internal projects mature into national-level grants and high-impact publications, the hospital's academic prestige is expected to grow, providing a significant long-term return on the initial internal investment and reinforcing the department's role as a strategic research hub.

## 5 Brand building and strategic value: from supportive service to center of excellence

### 5.1 Strategic repositioning: the precision diagnosis and therapy center

In today's competitive healthcare environment, a hospital's brand is increasingly defined by its ability to manage complex and refractory diseases. To translate the full value of nuclear medicine into a sustainable advantage, administrators could consider strategically repositioning the department from a traditional service unit to a multidisciplinary Precision Diagnosis and Therapy Center (3). This change is envisioned as more than a title update; it aims to formally recognize nuclear medicine as the hospital's “navigational hub” for oncology, cardiology, and neurology.

By promoting this center as a destination for high-acuity patients requiring precise molecular staging, the hospital seeks to capture a premium patient segment that values diagnostic certainty. This repositioning is intended to transform nuclear medicine from a mere cost center for imaging volume into a high-value strategic asset, which could support treatment precision and potentially prevent the substantial human and financial costs of ineffective therapies.

### 5.2 Operational synergy: the “One-stop” efficiency brand and TTD reduction

A strong brand is validated through patient experience and operational efficiency. Leveraging IoT and cloud-based platforms as discussed in Section 2, administrators could explore a One-Stop diagnostic-to-treatment workflow, where patients move from PET/CT scanning to multidisciplinary consultation within a single day. A key proposed administrative metric for this process is TTD, which measures the interval from initial clinical suspicion to MDT-approved treatment plan (14).

While reducing TTD is proposed as a mechanism to explicitly incorporate patient-centered outcomes by improving clinical experiences and increasing hospital throughput, this framework proposes it as a strategic goal for administrators to monitor. Such efficiency-driven brand aims to foster patient loyalty, ultimately contribute to a higher ROM by translating operational excellence into measurable institutional value.

### 5.3 Academic influence and international benchmarking

A top-tier hospital brand is ideally supported by academic excellence and global recognition. The establishment of Clinical-NM Liaisons and joint research funding is designed to support high-impact multidisciplinary publications, creating an internal culture of collaboration and innovation. Administrators should consider encouraging benchmarking against international standards, such as those from the Society of Nuclear Medicine and Molecular Imaging (SNMMI) and the European Association of Nuclear Medicine (EANM), and participation in multicenter trials.

Hosting regional forums on nuclear medicine management and contributing to international collaborations could generate a “halo effect” for the hospital, elevating the reputation of nuclear medicine as well as associated surgical and oncological departments. This academic-clinical synergy aims to contribute to the institution's status as a premier medical destination, potentially attracting elite talent and reinforcing the hospital's strategic positioning.

## 6 Implementation challenges and feasibility

### 6.1 Institutional stratification and resource scalability

The feasibility of this framework correlates with institutional scale and resource density. Large tertiary medical centers are ideally positioned to pilot the full suite of recommendations, such as IoT-driven resource leveling and dedicated Clinical-NM Liaisons. In contrast, smaller hospitals may face workforce and budgetary limitations that make specialized roles or physical satellite workstations less practical. For these institutions, a phased adoption approach prioritizing virtual MDT participation and basic protocol alignment over large-scale structural overhauls is suggested to ensure that core integration benefits are realized without overwhelming local resources. Specifically, the Clinical-NM Liaison duties could be temporarily performed concurrently by existing staff, and virtual consulting platforms could utilize existing telehealth infrastructure to bypass hardware constraints. Additionally, the TPI framework could be simplified into a basic compliance checklist within current administrative systems to maintain value-based oversight without extensive IT investment, thereby ensuring the framework's core strategic pillars remain scalable across all levels of healthcare institutions.

### 6.2 Regulatory, reimbursement, and technical barriers

The shift to value-based incentives may encounter regulatory hurdles in regions where hospital reimbursement remains strictly tied to fee-for-service models. Furthermore, the Clinical-NM Liaison role may face challenges regarding professional certification and billing rights in traditional administrative systems. These systemic barriers are often compounded by technical fragmentation, where the Hospital Information System (HIS), Nuclear Medicine Information System (NMIS), and Picture Archiving and Communication System (PACS) from different vendors are not fully interoperable. Addressing these challenges requires administrators to pilot internal incentive adjustments while investing in vendor-neutral platforms or custom middleware to bridge information silos.

### 6.3 Strategic change management and leadership

Perhaps the most significant barrier is the deeply ingrained siloed culture of medical specialties. Departments operating under traditional productivity-based incentives may resist the integration of nuclear medicine into established pathways, fearing a loss of clinical autonomy or shifts in resource allocation. Overcoming such resistance requires a shift in organizational culture driven by top-tier hospital leadership, such as the CEO or Vice President level. By fostering a shared vision that prioritizes collective patient outcomes over departmental territoriality, leadership can facilitate the collaborative environment necessary for the Management-Driven Integration Loop to thrive.

## 7 Conclusion

This paper proposes the Management-Driven Integration Loop as a conceptual strategic framework to bridge the gap between nuclear medicine's technological potential and its clinical impact. By aligning resource allocation, workflows, and performance incentives, hospital administrators strive to effectively reposition nuclear medicine from a supportive service to a central navigational hub for precision medicine. While successful implementation remains contingent on overcoming institutional, regulatory, and technical barriers, this integration is presented as a call to action for leadership to drive institutional excellence and improve patient outcomes through proactive, systematic governance.

## Data Availability

The original contributions presented in the study are included in the article/Supplementary Material, further inquiries can be directed to the corresponding author.
